# Cholangioscopy-guided recanalization of refractory bilioenteric occlusion

**DOI:** 10.1093/gastro/goag021

**Published:** 2026-03-14

**Authors:** Tao Han, Yun Cong, Yue Zhang, Yingchen Han, Shen Wu, Tingsong Chen

**Affiliations:** Department of Medical Oncology, The First Hospital of China Medical University, Shenyang, Liaoning, P. R. China; Department of Interventional Oncology, Shanghai Seventh People’s Hospital, Shanghai, P. R. China; Graduate School, Anhui University of Chinese Medicine, Hefei, Anhui, P. R. China; Department of Interventional Oncology, Shanghai Seventh People’s Hospital, Shanghai, P. R. China; Department of Medical Oncology, The First Hospital of China Medical University, Shenyang, Liaoning, P. R. China; Graduate School, Shanghai University of Traditional Chinese Medicine, Shanghai, P. R. China; Department of Interventional Oncology, Shanghai Seventh People’s Hospital, Shanghai, P. R. China

## Introduction

Benign bilioenteric anastomotic strictures occur in 3% to 13% of cases [1–3] and can progress to complete occlusion. When conventional percutaneous or endoscopic interventions fail, patients are often left with only two options: permanent external drainage or high-risk surgical revision [4, 5]. We reported two cases of complete bilioenteric anastomotic occlusion, defined by the absence of bowel opacification on cholangiography and failed cannulation by an experienced interventionalist. These cases were successfully managed with a novel technique. Using a percutaneous transhepatic approach, we performed direct recanalization under cholangioscopic guidance by applying controlled pressure to the occlusion’s center with a rigid guide-wire tip. The anastomosis was successfully traversed on the first attempt in both patients, without procedure-related complications. After subsequent balloon dilation and extended catheter stenting, both patients remained asymptomatic and free from reintervention for over 28 months. This cholangioscopy-guided recanalization technique offers a transformative, minimally invasive solution for an otherwise intractable clinical problem.

## Technical methods

External biliary decompression was initially established with a 10-Fr catheter in both patients. Definitive intervention was performed after percutaneous transhepatic biliary drainage (PTBD) tract maturation (typically 4–6 weeks post-drainage), providing a stable route for cholangioscope advancement. An electronic cholangioscope (SpyScope DS Access and Delivery Catheter M00546600) was introduced through the mature PTBD tract to provide continuous, high-resolution intraductal visualization. External biliary decompression was initially established with a 10-Fr catheter in both patients. Interventional cholangioscopy and definitive procedures were performed after tract maturation (typically 4–6 weeks post-drainage). The primary wire was a High Performance Glidewire (M00556581, 0.035-inch). Under direct cholangioscopic view, we first attempted to traverse the occlusion using the flexible hydrophilic tip. References to a ‘rigid tip’ denote the uncoated, stiffer proximal (core-support) segment of this same wire, used only after flexible-tip probing proved unsuccessful. When necessary, the rigid tip was advanced with controlled, steady axial pressure toward the center of the occlusion until traversal was achieved. Then, intestinal entry was confirmed by contrast injection under fluoroscopy. Following recanalization, the anastomosis was dilated the tract with a high-pressure balloon (Disposable Dilation Balloon, BDC-8/55–7/10), and a 10- or 12-Fr internal-external drainage catheter was left in place for prolonged stenting.

## Case 1

**Figure 1:**
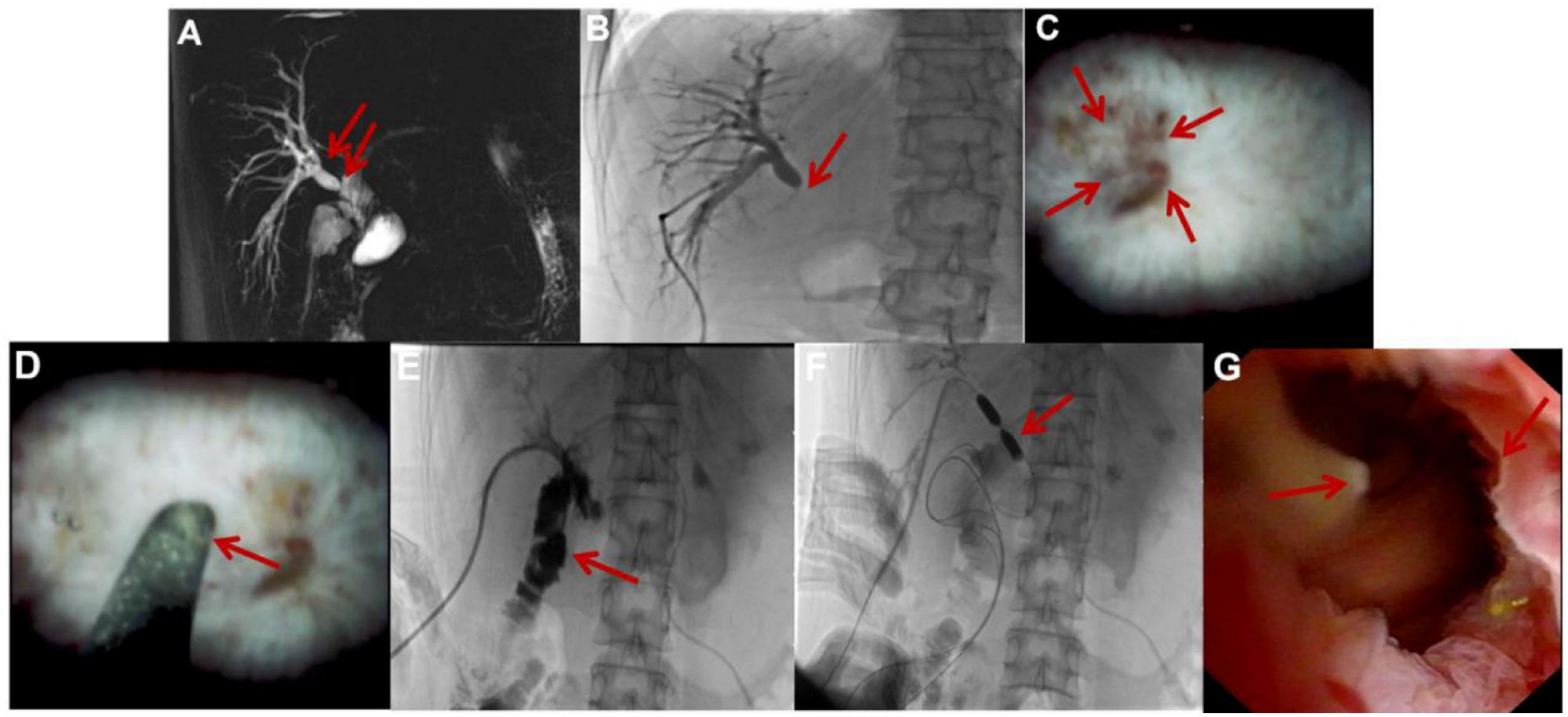
Recanalization of occluded bilioenteric anastomosis. (A) MRCP showing anastomotic stricture (arrow) with upstream dilation. (B) Cholangiogram showing non-opacification of bowel. (C) Cholangioscopic view of pinhole occlusion. (D) Initial attempts employed the flexible, hydrophilic tip of a 0.035-inch guidewire (Terumo Glidewire^®^) under continuous cholangioscopic vision. After sustained efforts exceeding 60 minutes yielded no progression—confirming a completely fibrotic, impassable occlusion—we transitioned to the rigid, uncoated proximal segment of the same wire. This segment was then advanced with steady, axial pressure to achieve controlled blunt dissection, guided by real-time visual and tactile feedback. Recanalization was judged by a tactile, definitive ‘give’ together with direct visual (and when appropriate, contrast) confirmation. Guide-wire tip advanced under direct vision. (E) Post-recanalization cholangiogram showing bowel opacification. (F) Balloon dilation of recanalized tract. (G) Follow-up cholangioscopy showing patent anastomosis.

A 33-year-old woman presented with recurrent cholangitis 1 month after Roux-en-Y hepaticojejunostomy for a congenital choledochal cyst. Magnetic resonance cholangiopancreatography (MRCP) suggested anastomotic stricture of the right hepatic duct (Fig. 1A). Percutaneous transhepatic cholangiography via an external drain demonstrated opacification of the right hepatic duct without bowel visualization (Fig. 1B), and all conventional fluoroscopy-guided attempts to traverse the occlusion failed. Consequently, we proceeded to cholangioscopy-guided recanalization. After consultation with our endoscopy team, balloon-assisted enteroscopy (BAE) based access was considered; however, it was either unsuccessful or deemed unlikely to achieve biliary cannulation because of the surgically altered anatomy. We therefore escalated to a percutaneous strategy and performed cholangioscopy-guided recanalization through the mature PTBD tract, which offered a short, stable, and controllable working channel for precise intervention. After successful wire traversal, definitive post-recanalization management was performed as described below. At our institution, cholangioscopy revealed a pinhole occlusion (Fig. 1C). The rigid tip of a guide-wire was advanced under direct vision toward the occlusion’s center, with a palpable ‘give’ confirming traversal (Fig. 1D). Contrast injection confirmed intestinal entry (Fig. 1E). Thereafter, to maintain patency following recanalization, balloon dilation (Fig. 1F) and catheter stenting for 1 year resulted in a patent, well-healed anastomosis on follow-up cholangioscopy (Fig. 1G). The patient has remained asymptomatic for 36 months. At the latest follow-up (30 June 2025), the patient remained clinically stable without adverse events or reintervention, with MRI/MRCP showing no biliary dilatation and labs (28 June 2025) indicating no cholestasis or systemic inflammation.

## Case 2

A 66-year-old woman with a complex surgical history, including left hepatic duct-jejunostomy for hilar stricture, had recurrent fever. MRCP showed isolated left-sided biliary dilation (Supplementary Fig. S1A). Percutaneous cholangiography confirmed left hepatic duct opacification without bowel visualization (Supplementary Fig. S1B); guide-wire cannulation failed. Given the patient’s complex post-reconstruction anatomy, we sought formal endoscopic input regarding a BAE-assisted approach. The endoscopy team advised that BAE was unlikely to provide reliable access (and any attempt would carry a low probability of successful traversal/cannulation). Accordingly, we proceeded with percutaneous cholangioscopy-guided recanalization via the established, mature PTBD tract, using this route as a direct and mechanically stable platform to enable controlled manipulation across the occlusion. Cholangioscopy revealed a pinpoint occlusion (Supplementary Fig. S1C). Guided recanalization with a rigid guide-wire tip successfully entered the bowel (Supplementary Fig. S1D and E), confirmed by contrast injection (Supplementary Fig. S1F). Once intraluminal access was confirmed, the anastomosis was balloon-dilated and then stented with an internal-external catheter for 6 months, resulting in durable patency. The patient remains well 28 months later. After cholangioscopy-guided recanalization, the patient experienced no procedure-related adverse events (e.g. bleeding, perforation, or cholangitis) and required no reintervention. Catheter exchange with adjunct balloon dilation was performed on 1 August 2025, and she was discharged in stable condition on 5 August 2025 (afebrile, no abdominal pain) with the drainage catheter capped and outpatient follow-up arranged. At the latest documented imaging follow-up (MRI/MRCP, 30 July 2025), postoperative changes with anastomotic narrowing and mild left intrahepatic duct dilatation were noted, without acute complications. Laboratory tests obtained around the same period showed a cholestatic pattern with minimal systemic inflammation.

## Discussion

We described the methodical application of a cholangioscopy-guided guidewire perforation technique to manage complete bilioenteric anastomotic occlusion in patients who have not responded to standard percutaneous and endoscopic approaches. The cholangioscope provides direct visualization of the occlusion, allowing precise targeting of its most vulnerable point—an advantage over blind fluoroscopic probing—and also serves as a stable conduit that transmits sufficient axial force to traverse dense scar tissue that would otherwise deflect a standard guidewire. Furthermore, using the guidewire’s rigid tip for controlled blunt dissection may minimize tissue injury compared with sharp instrumentation, potentially reducing the risks of perforation or hemorrhage. Safety was ensured by continuous cholangioscopic visualization combined with immediate, stepwise confirmation of intraluminal access (fluoroscopic behavior of the wire and low-volume contrast injection; with microcatheter/aspiration confirmation when needed), before proceeding to dilation and stenting.

Management of refractory bilioenteric occlusion remains challenging. In patients with surgically altered anatomy, balloon-assisted enteroscopy-assisted ERCP (BAE-ERCP) is often considered but can fail when access is limited or the anastomosis is completely occluded. When endoscopic access is not feasible, percutaneous approaches under fluoroscopy may be attempted, and combined ‘rendezvous’ strategies have been described; however, complete occlusion can still be difficult to traverse without direct intraductal visualization. Other salvage options reported for complete obstruction include EUS-guided biliary drainage/hepaticojejunostomy and magnetic compression anastomosis, which may require specialized expertise/devices and additional procedural complexity, and needle-knife or RF puncture techniques that raise concerns about tissue injury without reliable visualization. Against this backdrop, our technique advances care by leveraging an already mature PTBD tract to deliver high-resolution intraductal visualization and a mechanically stable working channel, enabling targeted, controlled traversal of the occlusion and subsequent dilation/stenting to maintain patency—while avoiding sharp cutting tools. Although limited to two patients, the durable patency and absence of procedure-related complications in these extreme cases support further evaluation in broader cohorts.

## Conclusions

Cholangioscopy-guided guide-wire recanalization offers a transformative minimally invasive solution for complete bilioenteric anastomotic occlusion. This approach enables precise, safe, and effective restoration of biliary-enteric continuity, liberating patients from permanent external drainage. The technique warrants consideration at specialized centers managing complex biliary complications.

## Supplementary Material

goag021_Supplementary_Data
