# Supplementary material for: Cholangioscopy-guided recanalization of refractory bilioenteric occlusion
Source: Gastroenterol Rep (Oxf). 2026 Mar 14;14:goag021. doi: 10.1093/gastro/goag021 (PMC12989147; doi:10.1093/gastro/goag021)
Supplement: goag021_Supplementary_Data [file goag021_supplementary_data.docx]

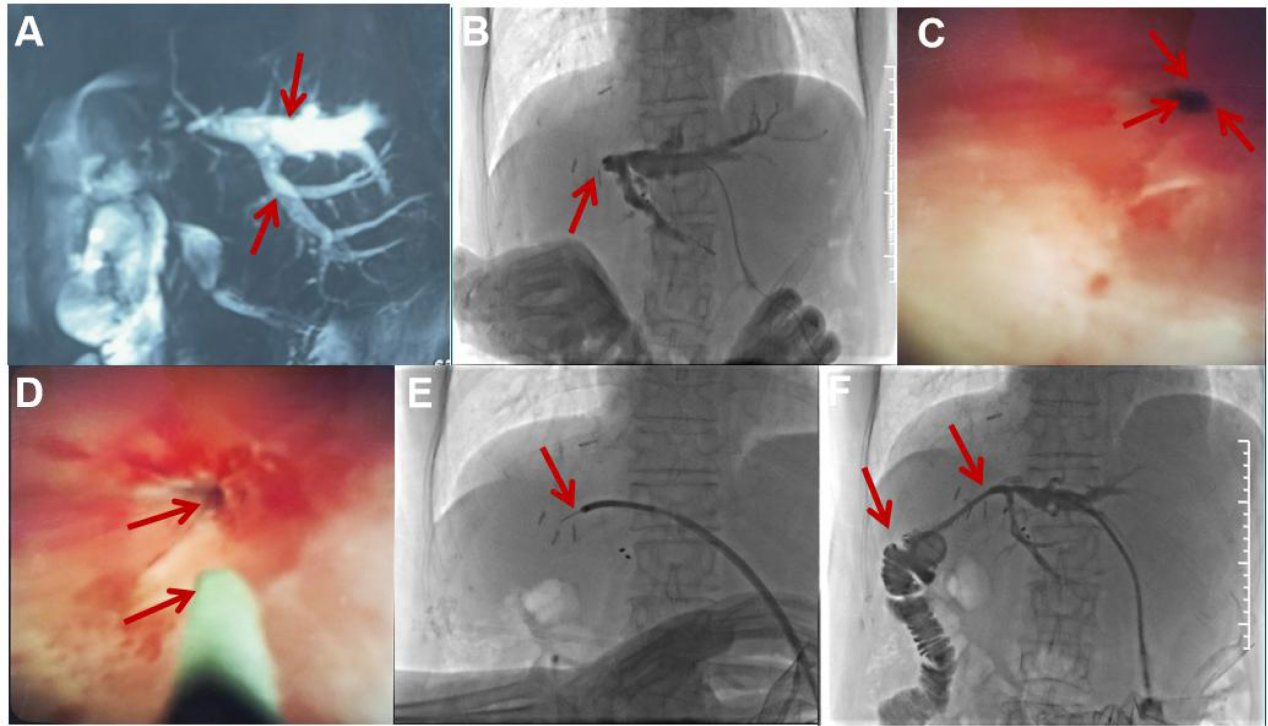


**Supplement Figure 1. Recanalization of Pinpoint Anastomotic Occlusion.**

(A) MRCP showing cutoff sign at anastomosis with left-sided dilation.

(B) Cholangiogram showing isolated left hepatic duct opacification.

(C) Cholangioscopic view of pinpoint occlusion.

(D) Guide-wire tip positioned at occlusion center.

(E) Fluoroscopy confirming wire traversal into bowel.

(F) Cholangiogram showing free contrast flow into bowel.
